# Association Between Early Hyponatremia and Clinical Outcomes in Critically Ill Patients: A Retrospective Cohort Study

**DOI:** 10.7759/cureus.56138

**Published:** 2024-03-14

**Authors:** Junya Itoh, Yoshitaka Aoki, Miki Omoto, Takayuki Katsuragawa, Soichiro Mimuro, Yoshiki Nakajima

**Affiliations:** 1 Department of Anesthesiology and Intensive Care Medicine, Hamamatsu University School of Medicine, Hamamatsu, JPN

**Keywords:** length of stay, renal replacement therapy, mortality, sodium, hyponatremia

## Abstract

Introduction: Hyponatremia, frequently encountered in intensive care (ICU) settings, plays a critical role in shaping patient outcomes. Despite its prevalence, contemporary research into its newly classified severity categories and their implications on mortality, renal function, and length of stay remains limited. This study aims to fill this gap by examining the impact of hyponatremia severity on these critical outcomes.

Methods: A retrospective analysis of ICU patients aged >18 years who were admitted between March 2019 and December 2022 was conducted at Hamamatsu University Hospital, Shizuoka, Japan. Patients who were readmitted or had incomplete data were excluded. Hyponatremia was categorized as mild (130-135 mmol/L), moderate (125-129 mmol/L), or severe (<125 mmol/L), following the criteria set by the European Society of Intensive Care Medicine. This classification utilized the lowest sodium concentration within 24 hours of ICU admission. The outcomes were in-hospital mortality, ICU mortality, newly implemented renal replacement therapy (RRT), and length of hospital and ICU stay. Outcomes were analyzed using multivariable logistic and linear regression models, adjusting for relevant covariates including age, sex, Acute Physiology and Chronic Health Evaluation (APACHE) III scores, and the use of mechanical ventilation.

Results: Of the 3,538 patients analyzed, 1,072 (30.3%) experienced hyponatremia: 894 (25.3%) mild, 144 (4.1%) moderate, and 34 (1.0%) severe. Multivariable analysis revealed no significant association between hyponatremia severity and in-hospital mortality rates across normonatremia (3.8%), mild (5.2%), moderate (11.8%), and severe (23.5%) groups, nor with ICU mortality. However, compared to normonatremia, moderate and severe hyponatremia were associated with increased RRT initiation (odds ratios = 3.83 and 6.36, respectively) and prolonged hospital stay (mean difference = 7.06 and 9.66 days, respectively), and ICU stays (mean difference, 1.02 and 2.70 days, respectively). Mild hyponatremia was not significantly associated with RRT or length of stay.

Conclusion: Moderate-to-severe hyponatremia did not influence mortality but was associated with increased RRT initiation and prolonged hospital and ICU stay. By contrast, mild hyponatremia was not associated with any clinical outcome. Further research is required to determine if correcting hyponatremia directly improves ICU patient outcomes, given the observational nature of the study.

## Introduction

Hyponatremia is one of the most prevalent electrolyte disorders, affecting 35-67% of hospitalized patients and 14-30% of intensive care unit (ICU) patients [1-3]. It is associated with an increased risk of adverse events in patients with underlying diseases and can lead to higher mortality in severe cases [2,4,5].

Studies have shown that patients with hyponatremia are more prone to developing acute kidney failure [6,7]. In addition, research has established a link between hyponatremia accompanied by acute kidney injury (AKI) and an increased risk of in-hospital mortality [4]. However, these reports utilized a traditional classification with a serum sodium concentration cutoff of 135 mmol/L, without focusing on the severity categories of hyponatremia.

In 2014, the European Society of Intensive Care Medicine and two other societies published clinical guidelines for the diagnosis and treatment of hyponatremia. These guidelines classify hyponatremia as mild (130-135 mmol/L), moderate (125-129 mmol/L), or severe (<125 mmol/L) [2]. However, recent reports on hyponatremia have rarely used this classification, adhering to the traditional cut-off of 135 mmol/L [8,9]. Consequently, the outcomes of the new categories of hyponatremia severity, especially their impact on renal function, remain unclear.

In this study, we focused on serum sodium levels within 24 hours of ICU admission and hypothesized that hyponatremia negatively affects clinical outcomes, such as mortality rate, renal function, and length of hospital stay, corresponding to the severity of the condition. We conducted a multivariable analysis using our ICU patient database to examine the relationship between the severity of hyponatremia and outcomes in critically ill patients.

## Materials and methods

Study design

This retrospective cohort study was conducted at Hamamatsu University Hospital (Shizuoka, Japan). The Ethics Review Board of Hamamatsu University School of Medicine approved the study protocol (approval number 23-137) and granted permission for the use of data for research purposes. Due to the retrospective design of the study and the absence of follow-up, the requirement for written informed consent was waived. The study aligned with the STROBE checklist and upheld the principles of the 1964 Declaration of Helsinki and subsequent amendments [10].

Patients and definition of hyponatremia

We included patients aged >18 years who were admitted to the ICU between March 2019 and December 2022. Patients who were readmitted to the ICU or had incomplete data were excluded. Our analysis focused on the lowest sodium levels within the first 24 hours of ICU admission. Sodium levels were classified into four groups according to the cutoffs outlined in the aforementioned guidelines: normonatremia, mild hyponatremia, moderate hyponatremia, and severe hyponatremia [2]. Most patients had a catheter in the radial artery for invasive blood pressure measurements from which blood gases were regularly drawn. Sodium measurements were performed using multiple blood gas analyses (with Radiometer ABL800 or ABL90 FLEX) or biochemical tests.

Outcomes

The outcomes included in-hospital mortality, ICU mortality, newly implemented renal replacement therapy (RRT), length of hospital stay, and length of ICU stay. Newly implemented RRT refers to patients who develop AKI during their ICU stay and require RRT initiation. Patients undergoing chronic hemodialysis upon ICU admission were excluded.

Statistical analysis

Categorical variables are presented as numbers and percentages, while continuous variables are presented as medians with interquartile ranges (IQRs). The baseline characteristics were analyzed using descriptive statistics. For each outcome, binary variables were examined using multivariable logistic regression analysis, and continuous variables were examined using multivariable linear regression analysis. The adjusted covariates included age, sex, body mass index, Acute Physiology and Chronic Health Evaluation (APACHE) III score, use of mechanical ventilation, AKI, administration of dopamine/norepinephrine, lactate levels, 2022 hospital admissions, chronic hemodialysis, and cardiac arrest prior to ICU admission. All statistical analyses, including the calculation of point estimates and 95% confidence intervals, were performed using Stata/MP 18 software (StataCorp, College Station, TX, USA).

## Results

**Figure 1:**
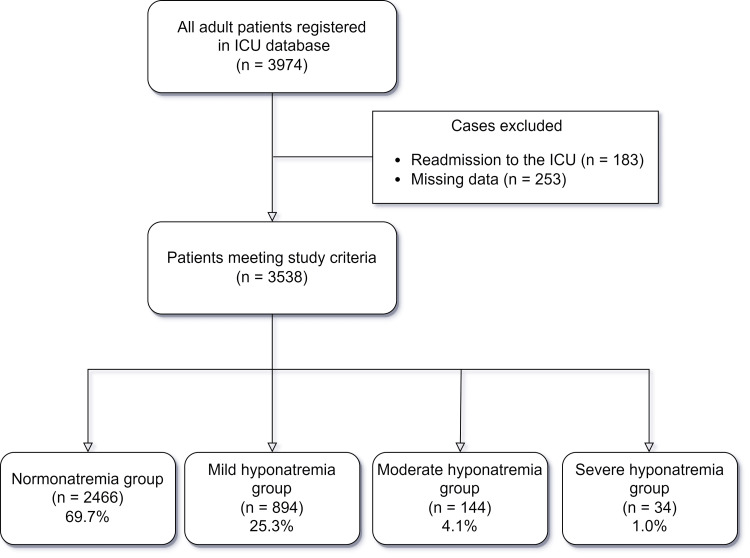
Flowchart of patient selection ICU: intensive care unit

During the study period, 3,974 patients were admitted to the ICU. We excluded 183 individuals for ICU readmission and 253 for missing data, leaving 3,538 in the dataset. Of these, 2,466 (69.7%) had normonatremia, 894 (25.3%) had mild hyponatremia, 144 (4.1%) had moderate hyponatremia, and 34 (1.0%) had severe hyponatremia (Figure 1).

Table 1 presents baseline characteristics and outcomes. The median lowest sodium value recorded within 24 hours was 136 (interquartile range (IQR) 134-138.3). The median age of the study group was 71 years (IQR, 60-78 years), and the patients were predominantly male (62.2%), with a median APACHE III score of 55 (IQR, 43-68). Cardiac arrest prior to ICU admission occurred in 2.5% of the patients, 24.4% were admitted in 2022, 40.2% required mechanical ventilation, and the median lactate level was 1.7 (IQR 1.1-2.8).

**Table 1:** Baseline characteristics and outcomes categorized by hyponatremia levels based on the lowest sodium concentrations BMI, body mass index; APACHE III, Acute Physiology and Chronic Health Evaluation III; ICU, intensive care unit; RRT, renal replacement therapy. Values are shown as number (%) or median (interquartile range).

	Total (n = 3,538)	Normonatremia (n = 2,466)	Mild hyponatremia (n = 894)	Moderate hyponatremia (n = 144)	Severe hyponatremia (n = 34)
Characteristics					
Sodium levels (mmol/L)	136 (134–138.3)	138 (136–139)	133 (132–134)	128 (127–129)	122.8 (120–124)
Age (years)	71 (60–78)	71 (59–78)	71 (61–78)	71 (64–79)	63 (50–74)
Male sex	2201 (62.2%)	1519 (61.6%)	566 (63.3%)	98 (68.1%)	18 (52.9%)
BMI (kg/m^2^)	22.2 (19.9–24.9)	22.6 (20.1–25.3)	21.5 (19.6–23.9)	21.2 (18.8–23.4)	20.0 (18.2–24.1)
APACHE III score	55 (43–68)	52 (40–65)	61 (49–74)	71 (62–90.5)	87 (62–110)
Chronic hemodialysis	265 (7.5%)	99 (4.0%)	135 (15.1%)	28 (19.4%)	3 (8.8%)
Cardiac arrest prior to ICU admission	89 (2.5%)	62 (2.5%)	17 (1.9%)	7 (4.9%)	3 (8.8%)
Admission year					
2019	763 (21.6%)	536 (21.7%)	188 (21.0%)	36 (25.0%)	3 (8.8%)
2020	934 (26.4%)	627 (25.4%)	255 (28.5%)	39 (27.1%)	13 (38.2%)
2021	978 (27.6%)	692 (28.1%)	239 (26.7%)	40 (27.8%)	7 (20.6%)
2022	863 (24.4%)	611 (24.8%)	212 (23.7%)	29 (20.1%)	11 (30.4%)
Use of mechanical ventilation	1421 (40.2%)	1016 (41.2%)	337 (37.7%)	50 (34.7%)	18 (52.9%)
Acute kidney injury	50 (1.4%)	24 (1.0%)	14 (1.6%)	8 (5.6%)	4 (11.8%)
Administration of dopamine	455 (12.9%)	323 (13.1%)	115 (12.9%)	14 (9.7%)	3 (8.8%)
Administration of norepinephrine	657 (18.6%)	375 (15.2%)	218 (24.4%)	50 (34.7%)	14 (41.2%)
Lactate (mmol/L)	1.7 (1.1–2.8)	1.7 (1.1–2.9)	1.7 (1.1–2.7)	1.6 (1.05–2.85)	2.55 (1.2–4.5)
Outcomes					
In-hospital mortality	165 (4.7%)	94 (3.8%)	46 (5.2%)	17 (11.8%)	8 (23.5%)
ICU mortality	52 (1.5%)	35 (1.4%)	12 (1.3%)	4 (2.8%)	1 (2.9%)
Newly implemented RRT	70 (2.1%)	34 (1.4%)	14 (1.8%)	14 (12.1%)	8 (25.8%)
Length of hospital stay (day)	21 (13–33)	19 (13–31)	23 (15–36)	26 (15–50)	40 (22–62)
Length of ICU stay (day)	2 (2–3)	2 (2–3)	2 (2–3)	3 (2–4)	4.5 (2–8)

**Figure 2:**
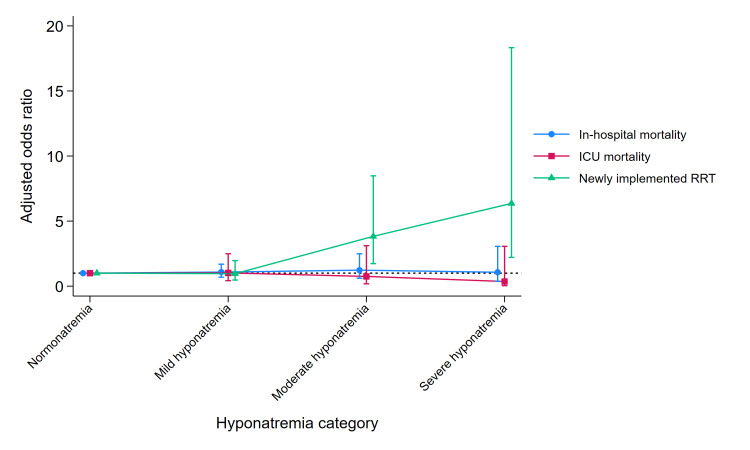
Clinical outcomes for binary variables The Y-axis represents the adjusted odds ratios from the multivariable analysis. The X-axis displays the categories classified according to sodium levels. The adjusted odds ratios are calculated using normonatremia as a reference value of 1. ICU: intensive care unit; RRT: renal replacement therapy

Figure 2 presents the multivariable analysis results for the outcomes related to the binary variables. No statistically significant differences were observed in the in-hospital or ICU mortality across the sodium concentration severity categories. However, the newly implemented RRT significantly increased the number of cases of moderate and severe hyponatremia compared with normonatremia.

**Figure 3:**
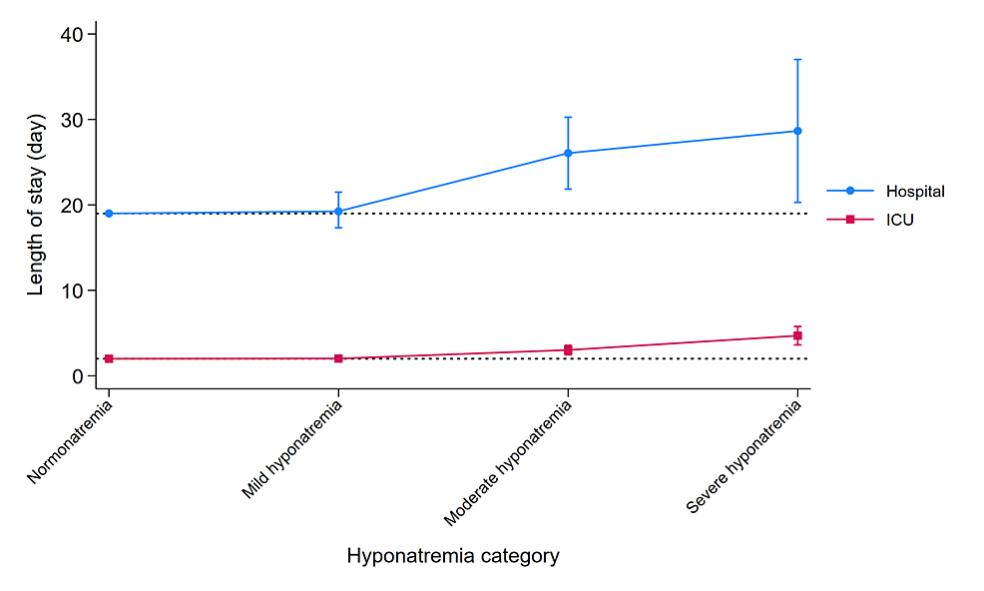
Clinical outcomes for continuous variables The Y-axis represents the length of stay, and the X-axis displays the categories classified by sodium levels. We established the median value of normonatremia (hospital stay: 19 days; ICU stay: two days) as a reference. ICU, intensive care unit

Figure 3 shows the multivariable analysis results for the outcomes associated with the continuous variables. In cases of moderate-to-severe hyponatremia, both the lengths of hospital stay and ICU stay were significantly longer than in cases of normonatremia.

Detailed data for these analyses are presented in Table 2.

**Table 2:** Results of multivariable analysis categorized by hyponatremia levels The results of the multivariable analysis are presented as OR with 95% CI and MD with 95% CI. OR: odds ratio; CI: confidence interval; ICU: intensive care unit; MD: mean difference; RRT: renal replacement therapy *P < 0.05

	Normonatremia (n = 2,466)	Mild hyponatremia (n = 894)	Moderate hyponatremia (n = 144)	Severe hyponatremia (n = 34)
In-hospital mortality, OR (95% CI)	1 (Reference)	1.08 (0.69 to 1.69)	1.23 (0.61 to 2.49)	1.07 (0.37 to 3.06)
ICU mortality, OR (95% CI)	1 (Reference)	1.02 (0.42 to 2.49)	0.75 (0.18 to 3.11)	0.36 (0.04 to 3.06)
Newly implemented RRT, OR (95% CI)	1 (Reference)	0.96 (0.47 to 1.96)	3.83 (1.73 to 8.48) *	6.36 (2.21 to 18.33) *
Length of hospital stay (day), MD (95% CI)	Reference	0.25 (-1.68 to 2.19)	7.06 (2.85 to 11.26) *	9.66 (1.30 to 18.02) *
Length of ICU stay (day), MD (95% CI)	Reference	0.02 (-0.23 to 0.26)	1.02 (0.48 to 1.56) *	2.70 (1.62 to 3.77) *

## Discussion

In our study, hyponatremia within 24 hours of ICU admission was observed in approximately 30% of the patients. However, mild hyponatremia, defined as sodium levels between 130 and 135 mmol/L, was not associated with poor clinical outcomes. Moderate-to-severe hyponatremia, with sodium levels below 130 mmol/L, was correlated with an increase in the need for new RRT and extended hospital and ICU stays. No significant association was observed between hyponatremia and increased in-hospital or ICU mortality.

The implications of our study lie in the categorization of hyponatremia according to clinical guidelines and establishment of its association with clinical outcomes. Previous studies have often investigated this condition based on a classification with a cutoff serum sodium level greater than 135 mmol/L [11-13]. Hyponatremia is a common clinical condition, and our findings suggest that mild cases do not require special care. In addition, previous studies have primarily assessed outcomes using the Kidney Disease Improving Global Outcomes criteria for AKI, with little exploration of the need for invasive RRT [4,5,14]. However, our study indicated a heightened risk of moderate or severe hyponatremia with the introduction of RRT.

Previous reports have shown that severe cases of hyponatremia are associated with increased mortality compared to those with normal sodium levels [15]. By contrast, our findings contradict those of earlier studies, indicating no correlation between hyponatremia severity and mortality rates. An observational study demonstrating increased overall mortality following cardiac surgery in patients with postoperative hyponatremia (24% in the hyponatremia group versus 18.2% in controls, p < 0.001) found that the hyponatremia group was significantly older and had lower left ventricular ejection fraction, higher mean pulmonary artery pressure, higher surgical risk, and more comorbidities [16]. This suggests a potential correlation between the occurrence of hyponatremia and the exacerbation of underlying factors, and in the present study, adjusting for the severity of underlying factors may have attenuated the association between hyponatremia and increased mortality. However, considering the significant differences observed in the newly implemented RRT, it can be inferred that the impact of low sodium levels on mortality was unlikely to be substantial.

Our study indicates that moderate to severe hyponatremia significantly affects the need for RRT and prolongs hospital and ICU stays. Hyponatremia primarily signifies fluid imbalance disorders that are common in critically ill patients in the ICU. Renal failure due to cardiorenal syndrome or sepsis can lead to reduced renal perfusion pressure, potentially reflected in serum sodium levels [17-19]. In addition, pathological evidence from past studies suggests a link between hyponatremia and chronic kidney disease, indicating potential underlying mechanisms connecting these conditions [20]. This suggests that serum sodium may serve as a predictive marker for the onset of AKI due to fluid imbalance in critically ill patients, potentially exacerbating factors, such as the length of hospital and ICU stay.

The present study has several limitations. First, acute hyponatremia is defined as a low sodium level that develops within 48 hours; however, our research focused on patients within 24 hours of ICU admission. The occurrence of acute hyponatremia (0.8%) is less frequent than chronic hyponatremia (6-29%) and may have a lesser impact on outcomes [21,22]. Second, we did not collect data on several factors affecting hyponatremia, including fluid imbalances, medications, presence of heart failure, arginine vasopressin levels, and urine osmolality [23,24]. Furthermore, vasopressin, a hormone that is closely linked to urine regulation, is involved in the development of hyponatremia. Assessing vasopressin levels could provide insights into the mechanisms underlying AKI development [25]. Third, although we considered severity and renal function as covariates, the possibility of unknown confounding factors that could not be adjusted for in the multivariable analysis cannot be ruled out. However, sodium levels are not modifiable factors and randomized controlled trials are not feasible, suggesting that observational studies will likely remain the main approach in the future. Finally, our study employed the lowest sodium concentration measurements from two types of blood gas analyzers and biochemical tests, acknowledging potential methodological variances [26]. We did not account for factors, such as proteins, hematocrit, and glucose, that could influence sodium measurements [27]. These aspects should be considered in future research.

## Conclusions

Hyponatremia within the first 24 hours of ICU admission was observed in approximately 30% of the patients. However, mild hyponatremia did not correlate with poor clinical outcomes. By contrast, moderate-to-severe hyponatremia was associated with an increased need for new RRT, as well as extended hospital and ICU stays. No significant association was observed between hyponatremia and increased in-hospital or ICU mortality. Further research is needed to explore clinical outcomes based on the severity of hyponatremia.
